# Low-Complexity Design and Validation of Wireless Motion Sensor Node to Support Physiotherapy

**DOI:** 10.3390/s20216362

**Published:** 2020-11-07

**Authors:** Jona Cappelle, Laura Monteyne, Jarne Van Mulders, Sarah Goossens, Maarten Vergauwen, Liesbet Van der Perre

**Affiliations:** 1 DRAMCO, Department of Electrical Engineering, Ghent Technology Campus, Katholieke Universiteit Leuven, B-9000 Ghent, Belgium; laura.monteyne@kuleuven.be (L.M.); jarne.vanmulders@kuleuven.be (J.V.M.); sarah.goossens@kuleuven.be (S.G.); liesbet.vanderperre@kuleuven.be (L.V.d.P.); 2 Department of Civil Engineering, Geomatics Section, Ghent Technology Campus, Katholieke Universiteit Leuven, B-9000 Ghent, Belgium; maarten.vergauwen@kuleuven.be

**Keywords:** physiotherapy, e-health, motion sensing, wireless charging, wireless connectivity, low power

## Abstract

We present a motion sensor node to support physiotherapy, based on an Inertial Measurement Unit (IMU). The node has wireless interfaces for both data exchange and charging, and is built based on commodity components. It hence provides an affordable solution with a low threshold to technology adoption. We share the hardware design and explain the calibration and validation procedures. The sensor node has an autonomy of 28 h in operation and a standby time of 8 months. On-device sensor fusion yields static results of on average 3.28° with a drift of 2° per half hour. The final prototype weighs 38 g and measures ø6 cm × 1.5 cm. The resulting motion sensor node presents an easy to use device for both live monitoring of movements as well as interpreting the data afterward. It opens opportunities to support and follow up treatment in medical cabinets as well as remotely.

## 1. Introduction

**Context: evolution in physiotherapy.** In the last few decades, physiotherapy has expanded from focusing on physical treatment solely with massage and stretching to a broader health context. Common treatments at a physiotherapist’s practice nowadays are for example post-operative rehabilitation, neurological injury treatment, occupational injury prevention, etc. Not only the field of application has developed, but also physical treatment techniques and approaches have improved, thanks to general medical progress. In particular, the technological improvements in imaging have helped physiotherapists for example to locate injuries more precisely and adjust the patient’s treatment [1]. The goal of the development reported in this paper is to introduce technological support at the patient’s side to improve the treatment, both curative and preventive.

**Focus: motion-sensing node.** In this paper, we present an Inertial Measurement Unit (IMU) sensor node to support the tracking and visualization of a patient’s execution of physical exercises or daily movements. The priorities for the sensor design were low-power, low-complexity, low-cost, and a small form factor. We achieved the goal to realize a sensor node with a diameter of maximum 6 cm, weighing less than 50 g, costing less than 30€, which can be lowered significantly for higher volumes. Considering the medical context, the sensor node must be hermetically sealed. Therefore, wireless charging is implemented. The measured data is transmitted wirelessly to a base station for further analysis. Calibration of the different sensors is done on-board to obtain measurements with higher precision than by using no calibration.

**Progress with respect to the state of technology.** Comparing the proposed sensor to currently available systems like [2,3], we focus on the raw output data rather than developing software that processes this automatically. Nonetheless, the raw data can be displayed graphically and present the data in a meaningful way. Secondly, the sensor design has features that contribute to the user-friendliness and accessibility for our target audience, the patient, and the physiotherapist. The presented motion sensor node thus exhibits a low complexity and user-friendly solution that can lower the cost with respect to available systems considerably, while preserving the same functionality. A smart watch, for example, is widely adopted to track overall activity of people. However, it is not fit to be attached anywhere on the body to monitor particular movements in physiotherapy, nor does it fulfill the low-cost and low-complexity requirements of the sensor nodes we aim for.

**Contribution.** We propose an innovative design, based on low-cost sensors, and the operation of the contactless sensor module, including automated calibration, which is in particular relevant to the targeted applications in e-treatment for physiotherapy. The novel contribution of this paper is threefold. First, we present the design and implementation of the wireless sensor node featuring wireless communication and charging and full filling the other requirements that were put forward. We share the open design it via GitHub [4]. Secondly, we elaborate on a simple, straightforward one-time sensor calibration procedure. This eases the operation of the system and ensures the reliable performance of the system. Lastly, we show how we performed the sensor validation with photogrammetry, which can be realized with inexpensive and widely available equipment in a real-life experiment. We further provide technological and application context.

**Structure of this paper**. This paper is further organized as follows: Section 2 presents the low-complexity design of the wireless sensor node. It zooms in on the calibration and wireless connectivity, as well as how the sensor node was optimized for low energy. The prototype is presented, meeting the initial requirements. In Section 3, the operation and accuracy of the sensor is validated using easily accessible equipment, avoiding expensive instruments. Next to this static validation, Section 4 elaborates on the dynamic behavior. This can be done with physical exercises. We explain the opportunities opened by the wireless sensor node for e-treatment in physiotherapy applications, and envisioned extensions to the system in Section 5. Section 6 summarizes the main conclusions of this paper and looks forward to potential future work.

## 2. Low Complexity Design of Wireless Motion Sensor Node

In the design of the sensor node, the following targets were set:

- **Accuracy.** The sensor node needs to be able to measure the human body movement with high precision. With proper calibration, it is possible to achieve a target accuracy of $\pm 2^{{}^{\circ}}$ with a sampling frequency of 50 Hz [5].
- **User-friendly.** The device needs to be easy to use, capable of being operated by anyone, regardless of any medical or technical background. We opted to implement wireless charging to increase user-friendliness in operation and maintenance. The data is also wirelessly transferred to eliminate a mess of cables and thus providing freedom of movement.
- **Autonomy.** Users want to focus on the application rather than constantly thinking about charging the device. Therefore, an autonomy of at least 5 h and a charge time of less than 1.5 h is necessary.
- **Affordable.** To provide an appealing multi-purpose product for a wide range of applications, it needs to come at a low cost. That way, we want to reach a wide audience, both professionals as individuals.

**Figure 1:**
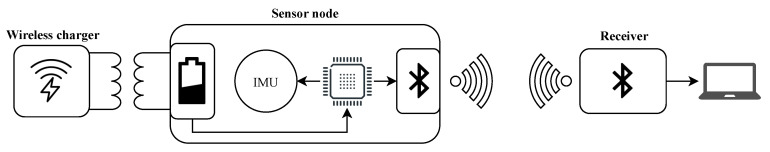
Overview of the hardware: The sensor node built around an Inertial Measurement Unit (IMU), wirelessly rechargeable and with wireless connectivity to a receiver base station.

The sensor node is built around an IMU. The data is wirelessly transmitted to a receiver and the internal battery can be wirelessly charged. Figure 1 shows an overview of the system. We discuss the main features of the sensor node here below.

### 2.1. Sensors

Motion can be monitored in several ways. A camera-based motion capturing system such as [6] can be used. These systems are highly accurate but expensive and cannot be used anywhere. Another method of monitoring movement that is more suited for our requirements, is by using an IMU. This type of sensor consists of several internal sensors. A 6 Degrees of Freedom (DoF) IMU is commonly used in recent works [7,8]. It has a constant drift in the resulting measurement data that cannot be corrected for. To eliminate this problem, our design uses a 9 DoF IMU in which the additional magnetometer provides a fixed reference. It consists of a Microelectromechanical Systems (MEMS) gyroscope, accelerometer, and compass. The IMU (ICM-20948 from Invensense ) [9] was chosen for its ultra-low power operating current and high accuracy. The gyroscope is set to ±2000 dps full scale, the accelerometer is set to ±4 G full scale and the magnetometer is set to ±4900 μT. The sample rate of all sensors is set to 50 Hz.

To obtain accurate orientation data, sensor fusion is needed. As used in [10], a Digital Motion Processor (DMP) can be very efficient for running specialized sensor fusion algorithms. By offloading computationally heavy calculations from the main processor, the system can be more power-efficient. The lack of control of the sensor fusion and calibration is a significant drawback. By implementing our own sensor fusion and calibration, we can implement the most suited fusion algorithms and have full control over the calibration. Some systems use sensor fusion algorithms like a Kalman filter [7], which provide very accurate results but can be computationally intensive. A complementary filter, which is very easy to process but typically provides less accurate results than a Kalman filter, is sometimes used. It uses a high pass filter for the gyroscope values and a low pass filter for the accelerometer values. This method of sensor fusion is inaccurate during long measurements with a lot of movement. In [8] for example, [8] use a complementary filter for measuring static angles, thus primarily depending on the accelerometer values. We need high dynamic accuracy with low processing power thus implemented a Madgwick filter [11], which combines the best of both worlds.

**Figure 2:**
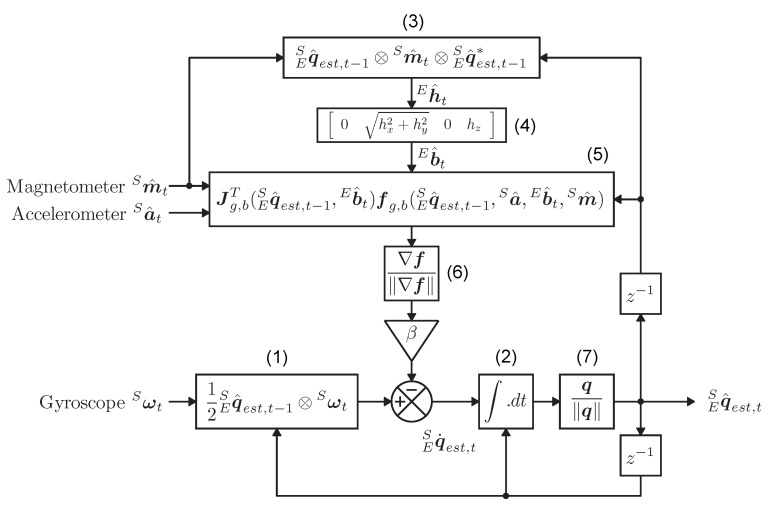
Block diagram Madgwick algorithm [11].

The algorithm runs on the central microcontroller (ARM Cortex M0+ microcontroller (EFM32HG) from Silabs) [12]. It is designed by [11]. By combining the efficiency of this algorithm with a high accuracy, a bit of battery power is saved. Quaternions, a very good way of representing orientations, are used for the calculations. Figure 2 illustrates the functional block diagram of this filter with ⊗ a quaternion product, $\dot{q}$ a quaternion derivative and $\hat{q}$ a normalized vector. The algorithm has two adjustable parameters, β and *f*. β represents the error on the gyroscope measurements as the magnitude of a quaternion derivative. It determines the proportion of the correction value for the gyroscope. *f* represents the frequency of the measurements. The orientation is mainly calculated by integrating the changes in angular velocity from the gyroscope (1). At the same time, an orientation is calculated using the accelerometer and magnetometer values. A gradient descent algorithm, represented by ▽, is used to find the most likely solution in the set of infinite solutions. (3) represents the measured orientation from the earth’s magnetic field. In (4), the measurements are normalized and mapped to the plane of the earth. In (5) = ▽f, the orientation from magnetometer and accelerometer values is calculated using a gradient descent algorithm. These values are normalized in (6) and used to correct gyroscope values with a factor β. These corrected gyroscope values are integrated in (2). Everything is further normalized in (7) to form unit quaternions and the results form is given as in Equation (1).

(1) $$\begin{array}{c} q=a+b\;\cdot \;i+c\;\cdot \;j+d\;\cdot \;k \end{array}$$

By using quaternions, we avoid the gimbal lock problem, the inability to uniquely represent an orientation, of Euler angles. When for example the pitch angle is 90°, yaw and roll cause the sensor to move in exactly the same fashion. Another problem is the inability to produce reliable estimates when an angle approaches 90° [13]. The benefit of the Madgwick algorithm is that we can run it at a very low speed and still get accurate results. At 50 Hz, the sample rate used by the sensor node, we get a static error of ±1° and a dynamic error of ±2° [11].

**Figure 3:**
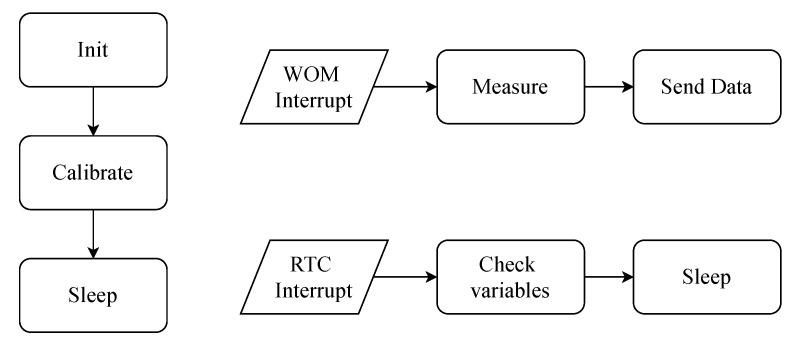
Flowchart code: The sensor node is calibrated once at initialization, a Wake On Motion (WOM) interrupt wakes up the system and measurements can start, an Real Time Counter (RTC) timer is used to periodically check the status of the sensor node to maximize autonomy.

The sensor node goes to sleep as much as possible to conserve battery energy. When the sensor node is picked up, the always-on accelerometer generates an interrupt and wakes up the system. Figure 3 illustrates this procedure. When the Madgwick parameters are set correctly, just a small portion of the accelerometer and compass values are used to correct the gyroscope error. When the sensor node wakes from sleep, the gyroscope has no reference orientation thus it would take approximately 30 s to obtain a correct orientation, depending on how much the actual orientation, when the sensor is picked up, differs from the orientation in sleep. After wake-up, the parameters of the Madgwick filter are dynamically adjusted to obtain a correct orientation quicker. The accelerometer and compass are used in the first few seconds of activity to obtain a correct reference frame. After this, the parameters are automatically adjusted to a high accuracy mode. In this mode, the integration of changes in angular velocity from the gyroscope is mostly relied on for calculating the orientation of the node. The accelerometer and the compass are only used to make small corrections.

As a low power design consideration, inactivity is detected by checking the gyroscope values every second in an interrupt service routine, called from an Real Time Counter (RTC) interrupt. The gyroscope values are supposed to be zero when idle. This procedure automatically puts the sensor node in sleep when it is not used.

### 2.2. Calibration

Calibration is an essential part of motion capturing systems. With calibration, the accuracy of the measurements can be drastically increased. In this design, a manual one-time calibration is used. The manual calibration allows the use of a very energy-efficient microcontroller and can yield calibration values with high accuracy. First, the gyroscope and the accelerometer are calibrated. This happens by simply putting the sensor node on a flat, leveled surface. During these measurements, no changes in angular velocity from the gyroscope or acceleration forces from the accelerometer are expected. The accelerometer and gyroscope are temporarily set to the most sensitive measurement range of ±250 dps full scale and ±2 G full scale to obtain the highest calibration accuracy possible. A few thousand measurements are taken by filling the First In First Out (FIFO) buffer of the IMU. From these measurements, a gyroscope and accelerometer bias offset is calculated and further subtracted from the actual measurements. After calibration of the gyroscope and accelerometer, the measurement ranges are changed back to ±2000 dps full scale and ±4 G full scale.

**Figure 4:**
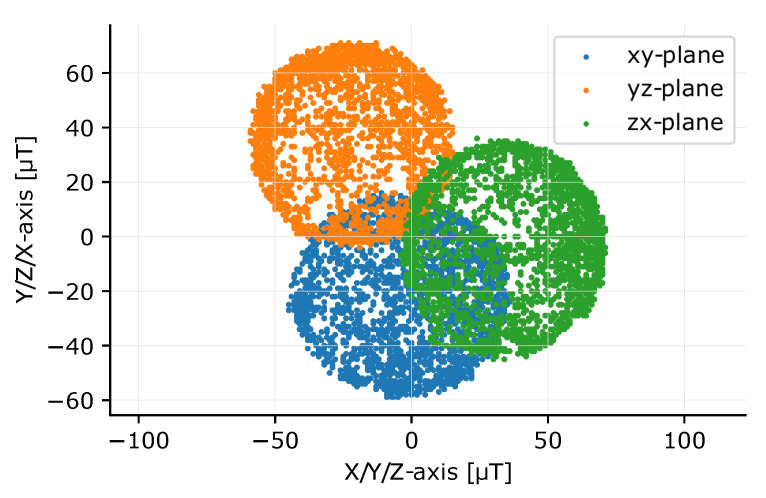
2D plot of a sphere after rotating the sensor node around each axis before calibration. The circles are not perfectly round (elliptical sphere in 3D) caused by soft iron distortions. Also, offsets between the centers of the circles and the origin, caused by hard iron distortions, are present.

**Figure 5:**
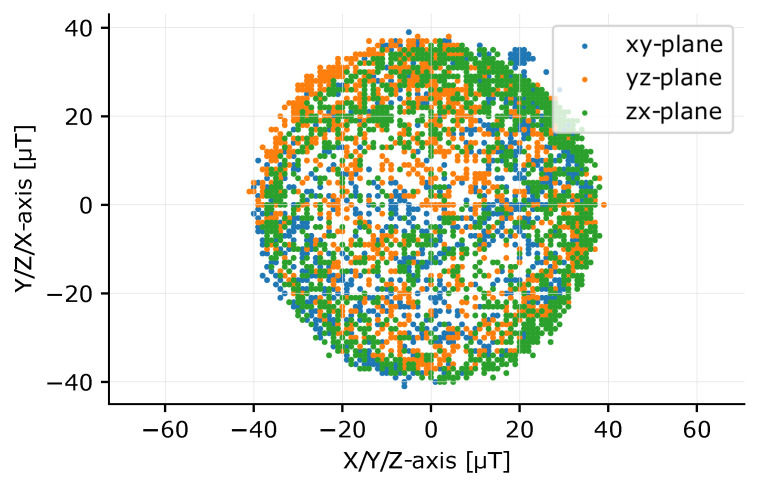
2D plot of a sphere after rotating the sensor node around each axis after calibration. The circles are perfectly round (near perfect sphere in 3D) and no offsets between the center of the circles and the origin are visible.

The compass is calibrated by rotating the device 360° around its three axes or performing a figure-8 movement. For these measurements, the maximum sampling frequency of 100 Hz is temporary used to have more data to work with and therefore obtain a better calibration. After calibration, the magnetometer sample rate is changed back to 50 Hz. The result of such a measurement is shown in Figure 4, a 2D visualization of the three planes of the 3D sphere after rotating the sensor node. Two types of distortions can occur on the IMU measurements: hard and soft iron distortions [14]. Hard iron distortions, caused by a permanent magnetic material, create a constant offset on the sphere. These offsets can be determined by calculating the center of the sphere and subtracting this value from the measurements. Soft iron distortions are caused by materials like iron. These materials do not create their own magnetic field but create a deformation on one or more axes. This will generally create an ellipse instead of a circle in a 2D plot. Soft iron distortions are more difficult to correct. Each axis is multiplied with a scale factor to calibrate the measurements. The minimal and maximal compass values captured in the calibration procedure of each axis are measured determined. The span of the compass values for all three axes is calculated, as well as the mean span for the three axes. The scale factor per axis is thus mean divided by the span of the axis that will be corrected. Equation (2) provides the equation for the x-axis scale factor, exemplary for the three axes. The result of these corrections, with the three circles perfectly round and centered, is given in Figure 5, showing that the calibration procedure operates correctly.

(2) $$\mathrm{Scale}\;\mathrm{factor}=\frac{max_{x}-min_{x}+max_{y}-min_{y}+max_{z}-min_{z}}{3\;\cdot \;(max_{x}-min_{x})}$$

### 2.3. Wireless Connectivity

Many wireless connectivity standards for Wireless Body Area Networks (WBAN) are available. We here briefly comment on the most considered technologies given the application focus of the presented design.

ZigBee operates with very low power usage. It works on top of the IEEE 802.14.4 standard, has a range of up to 100 m, and can be implemented as a mesh network. The low data rates of up to 250 kbps at 2.4 GHz make ZigBee less suited [15]. A second wireless standard is Z-Wave, a low data rate communication protocol with data rates of 40 kbps—100 kbps and a range of up to 30 m. Since it uses the 900 MHz band, it is not bothered by interference from 2.4 Ghz wireless communication like WiFi. It is commonly used in home automation for interconnecting energy efficient sensor nodes. The master-slave type network has a typical latency of 200 ms [16]. A third wireless standard is Bluetooth. It is based on the IEEE 802.15.1 standard, has a higher data rate of up to 2 Mbps and a range of up to 100 m. The more advanced Bluetooth protocol is widely used for data and audio transmission. It uses a master-slave model for communication [17]. For the design of the low power sensor node, Bluetooth Low Energy (BLE) is more appropriate. This special Bluetooth version is specifically designed for applications with very low power usage. A maximal data rate of 1 Mbps and a range of a few tens of meters can be achieved. BLE can use a master-slave model in a star topology or BLE devices can form a mesh network [16]. The advantage of BLE is its ability to directly connect to a smartphone or Bluetooth enabled device without the need for a separate receiving station. Following up with WiFi, based on the IEEE 802.11 standard with a very high data rate of 54 Mbps. The high power consumption makes WiFi less suited for a low power design [17]. We also studied the possibility of using a proprietary solution. The advantages are a possible further reduction in power consumption by packets with increased information density. Table 1 summarises the different wireless connectivity options in terms of power consumption, range, data rate and price. BLE is chosen for its low power consumption, sufficient range, relatively high data rate, low price, and high compatibility with existing devices.

A WBAN is necessary for transmitting the measured data. BLE is chosen for its high throughput, minimal power consumption, and interoperability with other devices [16]. The Proteus II module (AMB2623 module from WE based on an nRF52832) [18] is chosen for its small form factor and integrated PCB antenna. The data is transmitted at 0 dBm.

**Figure 6:**
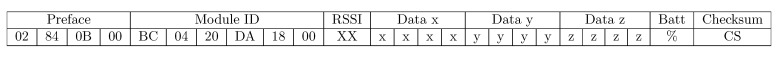
Bluetooth Low Energy (BLE) data packet structure: The data is composed of a preface, the module ID, three Euler angles, the remaining battery charge (percentage) and a checksum.

The data packet, sent out at 50 Hz, contains a preface, the module ID of the receiver, the RSSI, the data, and a checksum for error correction. This is clarified in Figure 6.

The quaternions from the Madgwick sensor fusion filter are converted to Euler angle floats. The three floats each take up four bytes in memory [19]. Exactly those bytes will be read from memory and transmitted wirelessly to ensure no loss in accuracy. One byte is added to transmit the battery status.

To guarantee a low power design, some software features are added. When the sensor node is picked up and cannot connect to a receiving device within five seconds, the sensor node enters sleep mode. The automatic reconnection of the sensor node with the receiving device is also built-in.

The receiving device is based on a development board (STM32L4+ microcontroller on an ST NUCLEO L45ZI development board) [20]. The same BLE module is chosen for this device. To be able to receive the transmitted data fast enough, an interrupt-based method is used together with a circular buffer [21]. The UART interrupt receives data and stores it in the buffer in the background. The received data is processed independently in the main program. A second UART transmits the data to a pc. A 3D representation of the orientation is written in VPython for visualization purposes.

### 2.4. Wireless Charging

Inductive wireless energy transfer is mainly used to recharge batteries of smartphones, wearables or, Internet of Things (IoT) devices. Implementation standards such as Qi, PMA, or AirFuel ensure a safe, efficient transfer of energy. Low power applications, below 5 W, often use e.g., proprietary solutions such as the “LinkCharge Low Power” technology from Semtech. Wearable devices, Electric toothbrushes, or LoRa based sensors are some of the many applications for the implementation of this technology [22]. ST Microelectronics also offers wireless power solutions for Smartwatches, or IoT battery-powered smart devices. The last option is to design your own Wireless Power Transfer (WPT) system without using existing standards. Building more efficient systems is time-consuming and not necessary since a lot of research has already been carried out in the 5 W WPT range.

In recent years, it has been generally accepted that the Qi is preferred over all other standards. The Wireless Power Consortium (WPC) manages and develops this standard. In the meantime, PMA, AirFuel and WPC have started a collaboration. All Qi-certified devices can communicate with each other. Charging a Qi-supported device can be performed by any Qi-certified charger. A series of functions in the standard ensures a safe charge cycle, such as thermal shutdown protection, foreign object detection, and overvoltage AC clamp protection [23].

The first wirelessly rechargeable smartwatches used proprietary WPT standards. New wearables switched to the Qi standard in contrast to wirelessly rechargeable smartphones, which were immediately equipped with the Qi standard. Recent smartphones are available with the option “Reverse Charging”, which means that the internal smartphone coil can be used to charge devices that support Qi [24]. This new feature offers the possibility of recharging smartwatches with a smartphone. It makes sense that Qi was chosen above all other options for the sensor module. In most households, a Qi charger or a smartphone that supports reverse charging is available. Future measurements with this sensor can be used within families, as they can recharge their sensor modules at home.

We here further discuss the actual implementation of the battery charging circuit in the design of the sensor node presented in this paper. Since energy is transferred wirelessly via the Qi protocol, a Qi receiver IC was used. A TI Qi receiver IC (BQ51050) [23] was selected because of its high efficiency, wireless power receiver, integrated rectifier, and battery charger in a single package. The BQ51050A variant, combined with a Li-Ion battery is chosen because of its 4.20 V output voltage limitation. It is paired with an inductor coil (760308101214 coil from WE) [25], chosen for its very small size and a relatively decent Q-factor. The charging current is 200 mA with a termination current of 20 mA to ensure fast and safe charging. Temperature control with automatic cut-off functionality at 60° is implemented by using a Negative Temperature Coefficient (NTC) resistor. Because of the small coil, we implemented some extra shielding to ensure a more optimal WPT.

**Figure 7:**
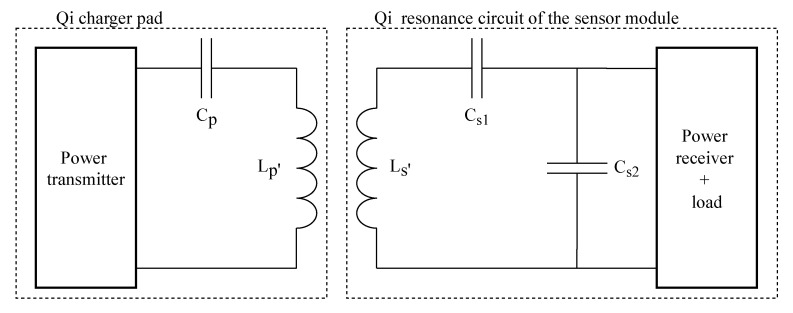
Wireless Power Transfer (WPT) setup. A Qi power transmitter with a Qi power receiver and load, based on LC resonant circuits.

Figure 7 shows the two coils in the system with corresponding resonant circuits. A power transmitter coil is present in each charger pad and a receiver coil in each battery-powered device. Wireless charging achieves higher link efficiencies when implementing LC resonant circuits on both the receiver and transmitter. The coupling factor between the two coils is very low. Therefore implementing a resonant circuit can filter out the leak inductance and improve the link efficiency drastically [26]. A Qi charger pad has a built-in amplifier connected to an LC series resonant circuit. The energy receiver side consists of an LC resonant circuit with L, $C_{s1}$, and $C_{s2}$. These capacities can be calculated with the Equations (3) and (4). $L_{s}^{\prime }$ represents the inductance measured when the receiver coil is placed on top of a charger pad. $L_{s}$ is the free-space inductance. $f_{s}$ and $f_{D}$ are fixed values respectively 100 kHz and 1 MHz [23].

(3) $$\begin{array}{cc} C_{1} & =\frac{1}{{\left(2\pi \;\cdot \;f_{s}\right)}^{2}\;\cdot \;L_{s}^{\prime }} \end{array}$$

(4) $$\begin{array}{cc} C_{2} & ={\left({\left(f_{D}\;\cdot \;2\pi \right)}^{2}\;\cdot \;L_{s}-\frac{1}{C_{1}}\right)}^{-1} \end{array}$$

Filling in the formula and converting to values for which actual hardware components are commercially available gives 100 nF for $C_{s1}$ and 1 nF for $C_{s2}$. Three other types of capacitors have an important function in the circuit. The BOOT, COMM, and CLAMP capacitors. The BOOT or bootstrap capacitors are used for driving the high-side FETs of the synchronous rectifier. The COMM capacitors allow communication with the charger pad. Here, capacitive load modulation is used. An extra capacitance is connected to the resonance circuit, which changes the resonance frequency. This change is visible on the charger pad side. Load modulation allows communication between the power receiver charging circuit and the power delivery pad circuit. Guidance values for resistive load modulation can be found in the datasheet. The CLAMP capacitors ensure overvoltage protection. Above the rectified voltage of 15 V, the CLAMP capacitors are switched to change the resonance frequency and protect the circuit against high voltages. The datasheet provides suggestions for these values. Values of 10 nF, 470 nF and 47 nF were used for the BOOT, CLAMP and COMM capacities, respectively [23].

### 2.5. Optimization for Low Energy

One of the main focuses of this work is the realization of a node with a convenient autonomy. A Li-Ion battery is chosen for its high energy density and low weight. The round battery with a capacity of 200 mAh is ideal for this prototype. This battery is rechargeable. With compatibility and ease of use in mind, Qi-compatible wireless charging is implemented. The whole system is powered at 2 V with an ultra-low Iq buck converter. In this configuration, a buck converter is much more efficient than a Low-dropout (LDO) regulator, even in sleep mode. The IMU works at 1.8 V. Here, the use of an LDO for the voltage drop of 0.2 V is more efficient. By running the whole system at 2 V instead of the traditional 3.3 V, a theoretical power difference of 9.610 mW is calculated when quiescent currents are neglected. This translates to a gain in the autonomy of 29.3 %. The sensor node consumes 0.102 mW in sleep mode and 25.839 mW in active measurement mode. This is reflected in an autonomy of 28 h in operation and of 261 days in sleep mode, which is well above the five hours put forward. An active power consumption of 25.839 mW is very low for this kind of system and can’t be significantly improved with the hardware we are currently using. This power consumption in combination with a 200 mAh battery allows for a long enough time between charges. The sleep current of 0.102 mW can possibly be improved by disabling the Qi-wireless charger completely when it’s not being used, thus eliminating quiescent currents. This can be done by using a MOSFET.

### 2.6. Prototype

**Figure 8:**
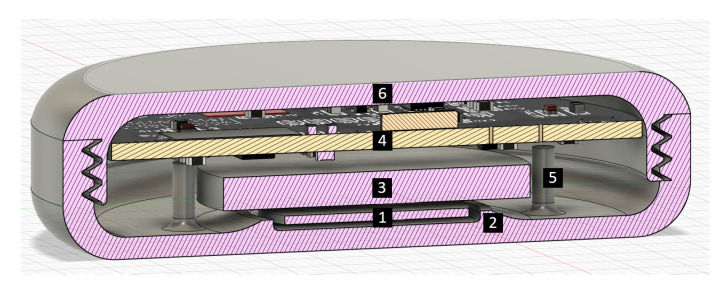
Cross-section of the sensor node. 1: wireless charging coil, 2: offsets, 3: battery, 4: PCB, 5: support pins, 6: twist top.

**Figure 9:**
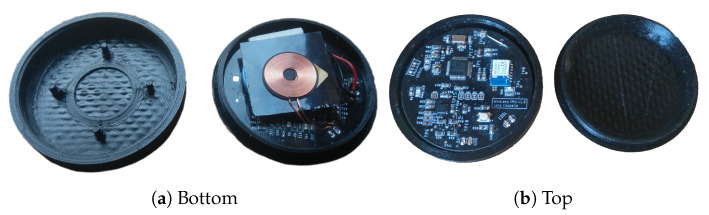
Result: sensor node in the 3D printed case. (**a**) Bottom side: wireless charging coil and battery. (**b**) Topside: IMU, microcontroller, and BLE chip.

A small physical design that is easy to place on the body is crucial. The sensor node features a round design with no sharp edges. The final prototype weighs 38 g and has dimensions ø6 cm × 1.5 cm. The structure of the case is shown in Figure 8. The wireless charging coil is positioned at the bottom (1). It is held in place by some offsets in the case (2). On top of that is the battery (3). Above the battery is the PCB (4) which is supported by four pins in the case (5). Everything is fastened nicely by the cover (6), which can be attached with a twist top. We did not yet hermetically seal the case for the initial experiments. By applying some sealant on the twist top, one can make the case more waterproof. Figure 9 shows the assembled prototype of the sensor node. The total cost of components is 28€ with case and 22€ without the case.

## 3. Validation with Easily Accessible Equipment

For the validation of the accuracy of the motion measurements realized by the sensor node, it is common to use professional equipment. The static verification process of the IMU has already been performed by using a computer monitored pan-tilt unit to place the sensor node in specific angles or by using a Vicon motion capturing system [6,8,27]. In the validation of the Madgwick filter for example, a Vicon motion capture system is also used [28]. Sensor validation on this equipment in general yields very accurate results but it is less accessible, expensive and time-consuming.

**Figure 10:**
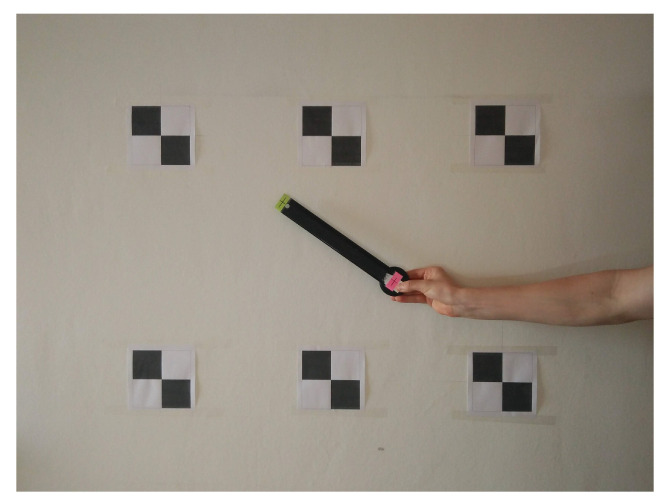
Method for sensor validation based on photogrammetry using convenient, commercial off-the-shelf equipment. By comparing the data from the IMU with the data extracted from images, the static error on the measurements can be derived for the pitch and roll axis.

We propose an alternative, very accessible way of validation using convenient equipment in the context of designing a low-cost system that is user-friendly. With photogrammetry, one can get a fairly accurate representation of the performance of the sensor node. In this method, we take and interpret photographic images of positions of the sensor. By comparing the data from the IMU with the data extracted from images, the static error on the measurements can be derived. The advantages are that this method can be performed almost anywhere and can be used with consumer off-the-shelf equipment. Since, in contrast to professional cameras, lower-cost equipment, such as a smartphone camera, suffers from lens distortions and lower quality recordings, some measures must be taken. To minimize the effect of the lower quality equipment, the camera is placed horizontally and perpendicular to the wall. This way, foreshortening effects are eliminated. Furthermore, the sensor is positioned such that its projection lies near the center of the image where radial distortion is minimal. This eliminates the need for a camera calibration procedure. Finally, we add several markers to the scene as shown in Figure 10. The relative position of these markers is measured up to ± 2 mm.

Since all we need is angles, we can perform measurements in the image and transfer them to the reference system of the sensor node. By attaching a lever to the sensor node, the accuracy of the readings in the image increases. The angle of the sensor can easily be measured by indicating front and endpoints of the lever (red and green points in Figure 10) and mapping these points in the image to points on the wall, using the coordinate system defined by the surrounding markers. By comparing the data from the IMU with the data extracted from the images, the static error on the measurements can be derived for the pitch and roll axis. In our experiments, only static measurements are performed. Dynamic measurements are possible as well, in which case video instead of images should be recorded and the video frames must be synchronized with the output data of the sensor node. Doing so, one can obtain angles at frame level. Instead of manually indicating points in each video frame, this process can be automated using image tracking [29,30].

**Figure 11:**
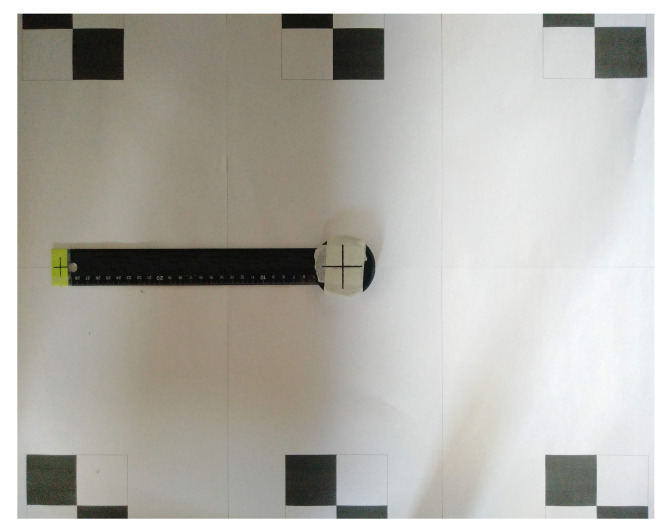
Photogrammetry-based method for yaw axis sensor validation.

Table 2 gives an overview of the measurements. For roll and pitch angles, the setup as shown in Figure 10 is used with the sensor node rotated 90° between roll and pitch measurements. Since the yaw values have no real fixed orientation, relative measurements are taken by using the setup as shown in Figure 11 where the sensor and markers are positioned on the floor instead of against the wall. Several static measurements were performed. The static sensor drift is 2° per half hour. The average error on the pitch axis is 3.06°, the average error on the roll axis is 2.75° and the average error on the yaw axis is 4.04°.

Alternatively, it is possible to measure all three (roll, pitch, yaw) angles at once by measuring the position of the lever endpoints in 3D using a stereo or multi-camera setup. However, drawbacks of such a method are the much higher complexity, the need for calibration and synchronization, and the lower accuracy in the depth dimension.

There are some irregularities in the measurements. The yaw value at 90° seems to be off. A root cause could be the influence of a nearby magnetic object. The sensor can get disturbed in the near proximity of magnetic objects such as speakers and smartphones. These magnets create a distortion in the magnetic field which isn’t fixed to the reference frame of the sensor node, thus can’t be corrected for in calibration. The user can perform reliable measurements when staying half a meter away from these objects to obtain accurate measurements. The pitch error at 90° is also too large. The reason is that Euler angles are not good at representing orientations in the neighborhood of 90° [13].

## 4. Validation with Real-Life Exercises

**Figure 12:**
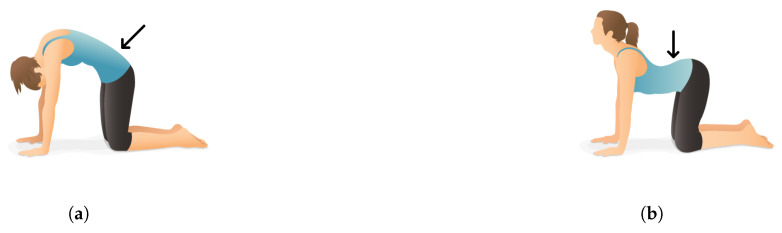
Illustration of the first exercise: periodic concavity of the spine (Images provided by Pocket Yoga (www.pocketyoga.com)). The arrows indicate the position of the sensor node. (**a**) Start position. (**b**) End position.Illustration of the first exercise: periodic concavity of the spine

To evaluate and validate the dynamic behavior of the sensor node and real-life operation, two back exercises are performed. The first exercise starts with a person kneeling with hands on the ground. The back is periodically rounded and made hollow, thus demonstrating the periodic concavity of the spine. This is illustrated in Figure 12.

**Figure 13:**
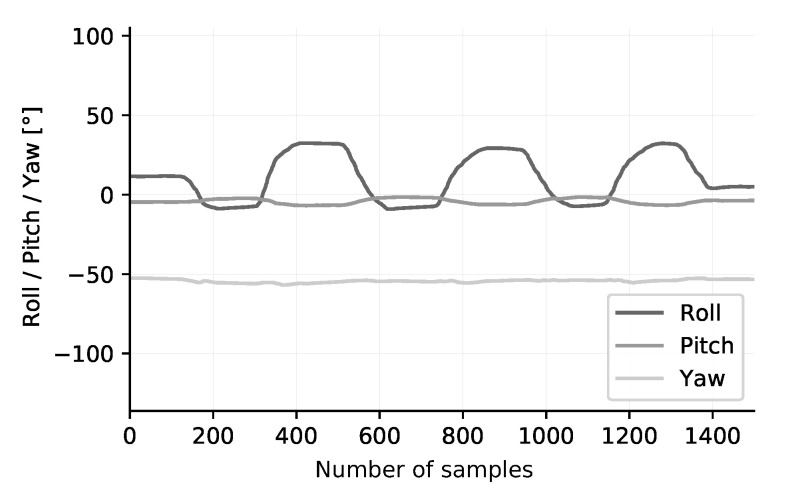
Exercise: Rounded back—hollow back. A periodic movement with a variation of ±45° on the roll axis can be observed. The pitch and yaw axis are stable.

**Figure 14:**
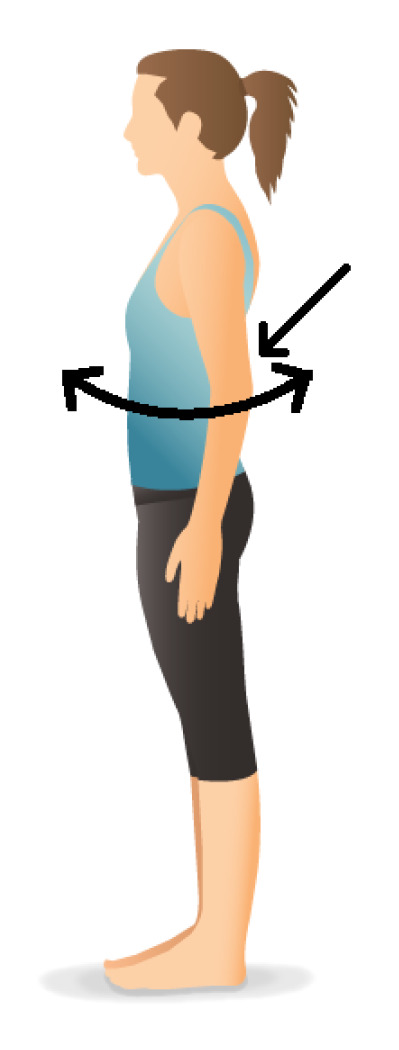
Illustration of the second exercise: lateral rotation of the back (Images provided by Pocket Yoga (www.pocketyoga.com)). The arrow indicates the position of the sensor node.Caption

**Figure 15:**
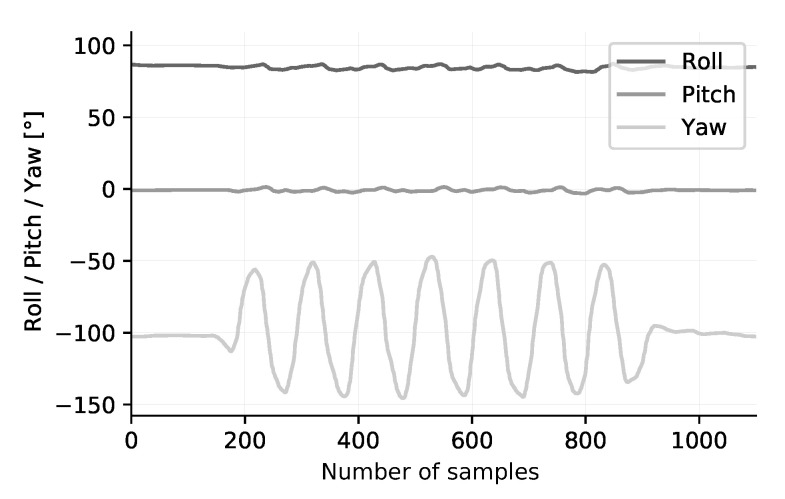
Exercise: Rotation of the back. A periodic movement with a variation of ±50° on the yaw axis can be observed. The roll and pitch axis are stable.

Figure 13 presents the result of the measurements. The exercise has been performed in a set of 3 repetitions. A periodic movement with a variation of ±45° on the roll axis can be observed. The pitch axis shows a little bit of sideways rotation in the lower back. The yaw axis is stable, which is to be expected. A second captured exercise is the lateral rotation of the back, illustrated in Figure 14. The patient should rotate the hull sideways, while maintaining stable lower limbs. The measured result is represented in Figure 15. An angular deviation of ±50° is present in the yaw axis data. Small changes in roll and pitch values are also observed. These two exercises provide a first evaluation of the dynamic characteristics of the sensor node. We clearly see that the amount of samples taken is appropriate to acquire accurate results. However, more testing, either by dynamic photogrammetry or with specialized equipment, is needed before a firm conclusion on accuracy can be made.

## 5. Opportunities in e-Treatment Applications and Extended Functionalities

We here first explain the opportunities opened up by stand-alone low-cost and low-complexity sensor nodes in physio-therapeutic e-treatment. We benchmark the current solution and introduce further extensions of the system that can bring interesting features for both private and professional users.

### 5.1. Opportunities in Supporting e-Treatment in Physiotherapy

The presented wireless sensor node has been designed to meet the particular needs to support physiotherapy treatment. We wish to introduce technical support at the patient’s side to improve both curative and preventive treatment. The sensor thus enables *e-treatment*, which we define as (remote) physical therapy that is supported by measurements made by wireless sensors. In a curative treatment, the patient can wear the sensor to assist the physiotherapist in the evaluation of (eventual take-home) rehabilitation exercises. A preventive treatment could consist of monitoring a person’s daily movements or measuring a patient’s flexibility. We specifically expect measurements at work to be interesting, knowing that the large majority of neuromusculoskeletal disorders result from repetitive movements and bad posture at work [31].

Also important in our definition of e-treatment, is the word *remote*. In the case of remote treatment, the patient is not physically present in the physiotherapist’s practice, but for example at home and possibly assisted with one or more sensors. Especially because of the increasing cost of healthcare in our ageing society, it is important to look at efficient and low-cost alternatives. The connection is then real-time through a conference call, or non real-time by exchanging exercises over a manual for example. There are several reasons why a remote session can be preferred over a conventional consultation:

- The patient can perform the session more or less independently.
- The patient is abroad and wants to continue the treatment with the same physiotherapist. For example, elite athletes who have to travel a lot.
- A patient is not allowed to leave the house. The COVID-19 pandemic proved this to be a realistic scenario.

The effectiveness of e-treatment in a remote sense is exhaustively discussed in [32]. A last important field of application is the education of physiotherapists. With the help of our technology in a bigger ecosystem, we want to reach physiotherapist with e-learning and help them train and improve. In summary, the sensor can enable remote treatment, as well as support conventional consultations or even acquire measurement data for preventive purposes.

### 5.2. Extension to Multiple Sensor Nodes

Richer information and support in rehabilitation and e-treatment could be offered by the combination of multiple sensor nodes, either of the same type or using heterogeneous sensors. An especially relevant type is an Surface Electromyography (sEMG) sensor module for measuring muscle activity. While we have designed the first prototype for this sensor type, in a future version we will combine the IMU and the sEMG sensor into one module. By combining these sensors, we can capture a more complete picture of what the human body is doing. However, this generates extra technological challenges, especially with respect to synchronization, both intra- and inter-module, required to ensure concurrent measurements. Synchronization between the sEMG and the IMU can be implemented using a shared clock. Both sensors will experience the same clock drift. BLE beacon packets from a central node, in this case the receiver, or a custom protocol can be used to synchronize the clocks between sensor nodes [33]. The data can be transmitted using unidirectional beacon packets without re-transmission. This type of data transfer is very simple but does not guarantee the packet arrives at the receiver. A better way would be to use the BLE re-transmission functionality to ensure the packets are received properly. Time synchronization beacon packets could be sent in between. It is evident that both the electrical and the mechanical design will be more complicated, not in the least because of the need to integrate the functions in a small space.

## 6. Conclusions and Future Work

**Conclusion.** In this paper, a wireless on-body sensor node for measuring movement is presented. The careful choice of components, software optimizations, and overall low power design considerations lead to a sensor node with an autonomy of 28 h. An ‘always-on’ buttonless design, with a standby time of 8 months is developed that is ready to measure whenever it is picked up. We explained the calibration of the sensor node and zoomed in, in particular on a photogrammetric procedure to validate the sensor with easily accessible, low-cost equipment. On-device sensor fusion by using a Madgwick filter yields static results of on average 3.28° with a drift of 2° per half hour. The final prototype weighs 38 g and measures ø6 cm × 1.5 cm. The result of this work can be used in a broad range of applications. It allows doctors and physiotherapists to have an easy to use device to pass along with patients and afterward interpreting the results, it can be used for live monitoring of rehabilitation exercises or anything motion tracking related.

**Future work.** We see multiple opportunities in future work to both the current sensor node, and to extend it with new functionality and features. Firstly, we plan to further examine the accuracy of the sensor node by checking it against specialized equipment. We will add other sensors to get a more in-depth view of the human body. We also designed a sEMG sensor for measuring muscle activity. These two sensors could be integrated into one module to perform simultaneous measurements. Synchronization, both inter- and intra-sensor node, will be implemented to ensure precise, simultaneous measurements. A future upgrade could also implement a real-time calibration by using artificial intelligence [34]. This could well be implemented on a low power microcontroller with an ARM Cortex M4 chip (nRF52832 from Nordic Semiconductor), which is already used in the BLE module. By running the Bluetooth stack and the peripheral code on the same chip, we could eliminate the central Cortex M0+ microcontroller and further reduce the power consumption. We could also design our own PCB antenna. In the current design, the data is, other than being visualized, not further processed. To detect and analyze complex movements, further data analysis as well as learning algorithms can be implemented. Another extension to the system is a direct communication between the sensor nodes and a smartphone through an app. This eliminates the need for a separate receiver.

## Figures and Tables

**Table 1:** Comparison between available wireless technologies: ZigBee, Z-Wave, Bluetooth 5, BLE, and WiFi [15,16,17].

	ZigBee	Z-Wave	Bluetooth 5	BLE	WiFi
Power consumption (max)	100 mW	1 mW	100 mW	10 mW	>100 mW
Range (max)	100 m	30 m	100 m	<100 m	1000 m
Data rate (max)	250 kbps	100 kbps	2 Mbps	1 Mbps	54 Mbps
Price	Low	High	Very low	Very low	Average

**Table 2:** Result of pitch, roll, and yaw static measurements with their respective error at different angles.

	Target Angle [°]	Reference [°]	Sensor [°]	Error [°]
**Pitch**	0	0.08	−3.2	3.28
	45	44.76	42.5	2.26
	90	90.19	95.04	−4.85
	180	178.45	176.6	1.85
**Roll**	0	0.47	1.8	−1.33
	45	48.41	44.8	3.61
	90	90.15	87	3.15
	180	180.01	177.1	2.91
**Yaw**	45	48.03	45.1	2.93
	90	95.49	88.9	6.59
	180	182.18	185.2	−3.02
	270	274.12	270.5	3.62

## Abbreviations

The following abbreviations are used in this paper:

BLE	Bluetooth Low Energy
DMP	Digital Motion Processor
DoF	Degrees of Freedom
FIFO	First In First Out
IMU	Inertial Measurement Unit
IoT	Internet of Things
LDO	Low-dropout
MEMS	Microelectromechanical Systems
NTC	Negative Temperature Coefficient
RTC	Real Time Counter
sEMG	Surface Electromyography
WBAN	Wireless Body Area Networks
WOM	Wake On Motion
WPC	Wireless Power Consortium
WPT	Wireless Power Transfer

## References

[1] Porciuncula F., Roto A.V., Kumar D., Davis I., Roy S., Walsh C.J., Awad L.N. (2018). Wearable Movement Sensors for Rehabilitation: A Focused Review of Technological and Clinical Advances. PM&R.

[2] Inertial Motion Capture System|Shop|Eliko. https://www.eliko.ee/shop/inertial-motion-capture-system/.

[3] IMU Sensor Development Kit|Wireless IMU Sensor|9DOF Motion Sensor. https://www.shimmersensing.com/products/shimmer3-development-kit.

[4] Cappelle J. Wireless Motion Sensor Node. https://github.com/DRAMCO/NOMADe-Wireless-Motion-Sensor-Node.

[5] Brodie M., Walmsley A., Page W. (2008). The static accuracy and calibration of inertial measurement units for 3D orientation. Comput. Methods Biomech. Biomed. Eng..

[6] Vicon|Award Winning Motion Capture Systems. https://www.vicon.com/.

[7] Kadir K., Yusof Z.M., Rasin M.Z.M., Billah M.M., Salikin Q. Wireless IMU: A Wearable Smart Sensor for Disability Rehabilitation Training. Proceedings of the 2018 2nd International Conference on Smart Sensors and Application (ICSSA).

[8] Petropoulos A., Sikeridis D., Antonakopoulos T. (2020). Wearable Smart Health Advisors: An IMU-Enabled Posture Monitor. IEEE Consum. Electron. Mag..

[9] ICM-20948—TDK. https://www.invensense.com/products/motion-tracking/9-axis/icm-20948/.

[10] Fei Y., Song Y., Xu L., Sun G. Micro-IMU based Wireless Body Sensor Network. Proceedings of the 33rd Chinese Control Conference.

[11] Madgwick S.O.H., Harrison A.J.L., Vaidyanathan R. Estimation of IMU and MARG orientation using a gradient descent algorithm. Proceedings of the 2011 IEEE International Conference on Rehabilitation Robotics.

[12] EFM32 Happy Gecko Family EFM32HG Data Sheet. https://www.silabs.com/documents/public/data-sheets/efm32hg-datasheet.pdf.

[13] Understanding Euler Angles—CH Robotics. http://www.chrobotics.com/library/understanding-euler-angles.

[14] Tuupola M. How to Calibrate a Magnetometer?. https://appelsiini.net/2018/calibrate-magnetometer/.

[15] Ergen S. (2004). ZigBee/IEEE 802.15.4 Summary. UC Berkeley Sept..

[16] Bin Ab Rahman A. Comparison of Internet of Things ( IoT ) Data Link Protocols. https://www.semanticscholar.org/paper/Comparison-of-Internet-of-Things-(-IoT-)-Data-Link-Rahman/1cf94e2ebb27aaecdae3742e444ca9e87314216b.

[17] Danbatta S.J., Varol A. Comparison of Zigbee, Z-Wave, Wi-Fi, and Bluetooth Wireless Technologies Used in Home Automation. Proceedings of the 2019 7th International Symposium on Digital Forensics and Security (ISDFS).

[18] Proteus-II—Bluetooth Smart 5.0 Module (AMB2623). https://katalog.we-online.de/en/wco/WIRL_BTLE_5.

[19] Cx51 User’s Guide: Floating-Point Numbers. http://www.keil.com/support/man/docs/c51/c51_ap_floatingpt.htm.

[20] NUCLEO-L4R5ZI—STM32 Nucleo-144 Development Board with STM32L4R5ZI MCU— STMicroelectronics. https://www.st.com/en/evaluation-tools/nucleo-l4r5zi.html.

[21] UART Receive Buffering—Simply Embedded. http://www.simplyembedded.org/tutorials/interrupt-free-ring-buffer/.

[22] Semtech Releases Next-Generation LinkCharge^®^ LP (Low Power) Wireless Charging Platform. https://www.semtech.com/company/press/semtech-releases-next-generation-linkcharge-lp-low-power-wireless-charging-platform.

[23] BQ5105xB High-Efficiency Qi v1.2-Compliant Wireless Power Receiver and Battery Charger. ti.com/lit/ds/symlink/bq51051b.pdf?ts=1604672421028.

[24] Galaxy S10 Reverse Wireless Charging Feature: How to Use It. https://www.valuewalk.com/2019/03/galaxy-s10-reverse-wireless-charging/.

[25] WE-WPCC Wireless Power Charging Receiver Coil 760308101214. https://www.we-online.com/catalog/datasheet/760308101214.pdf.

[26] Puers R. (2009). Inductive Powering: Basic Theory and Application to Biomedical Systems.

[27] Computer Controlled Pan-Tilt Unit Model PTU-D46. www.imagelabs.com/wp-content/uploads/2011/01/Specs-PTU-D46.pdf.

[28] Madgwick S.O.H. (2010). An Efficient Orientation Filter for Inertial and Inertial/Magnetic Sensor Arrays.

[29] Shi J., Tomasi C. Good Features to Track. Proceedings of the IEEE Conference on Computer Vision and Pattern Recognition.

[30] Isard M., Blake A. (1998). CONDENSATION—Conditional Density Propagation for Visual Tracking. Int. J. Comput. Vis..

[31] de Kok J., Vroonhof P., Snijders J., Roullis G., Clarke M., Peereboom K., van Dorst P., Isusi I. (2019). Work-Related Musculoskeletal Disorders: Prevalence, Costs and Demographics in the EU.

[32] Dierick F., Buisseret F., Brismée J.M., Fourré A., Hage R., Leteneur S., Monteyne L., Thevenon A., Thiry P., Van der Perre L. Opinion on the Effectiveness of Physiotherapy Management of Neuro-Musculo-Skeletal Disorders by Telerehabilitation. https://www.ifompt.org/site/ifompt/Telerehab_EN.pdf.

[33] Coviello G., Avitabile G., Florio A. (2020). A Synchronized Multi-Unit Wireless Platform for Long-Term Activity Monitoring. Electronics.

[34] Claesson E., Marklund S. (2019). Calibration of IMUs using Neural Networks and Adaptive Techniques Targeting a Self-Calibrated IMU. Master’s Thesis.

